# Predictive Value of Direct Disk Diffusion Testing from Positive Blood Cultures for Detection of Antimicrobial Nonsusceptibility

**DOI:** 10.3390/microorganisms13020398

**Published:** 2025-02-12

**Authors:** Tammy Ting-Yan Wong, Chung-Ho Lee, Hester Wing-Sum Luk, Cindy Wing-Sze Tse, Pak-Leung Ho

**Affiliations:** 1 Department of Clinical Pathology, Kwong Wah Hospital, Hospital Authority, Hong Kong, China; wty469@ha.org.hk (T.T.-Y.W.); lch101@ha.org.hk (C.-H.L.); ws.luk@ha.org.hk (H.W.-S.L.); tsewsc@ha.org.hk (C.W.-S.T.); 2 Department of Microbiology, Queen Mary Hospital, University of Hong Kong, Hong Kong, China; 3 Carol Yu Centre for Infection, University of Hong Kong, Hong Kong, China

**Keywords:** Enterobacterales, bacteremia, antimicrobial resistance, Hong Kong

## Abstract

Antibiotic resistance poses a significant global threat, particularly in the context of bloodstream infections. Early antimicrobial susceptibility testing plays a crucial role in guiding clinicians to optimize treatment and enhance patient outcomes. Direct disk diffusion testing (dDDT), utilizing positive blood culture broth as an inoculum, provides results one day earlier than the standard method using bacterial colonies. This retrospective study evaluated the ability of dDDT to predict nonsusceptibility to commonly used antibiotics. From January 2021 to December 2023, a total of 1473 blood cultures positive for a single pathogen (Enterobacterales, *Pseudomonas aeruginosa*, *Staphylococcus aureus*, β-hemolytic streptococci, or *Enterococcus* spp.) were examined. The results of dDDT were compared against the standard disk diffusion method as the reference standard. A total of 9754 organism–antibiotic pairs were analyzed. The positive predictive values were more than 98% for clinically significant resistant phenotypes, including ceftriaxone, ceftazidime, cefepime, and meropenem nonsusceptibility in Enterobacterales, ceftazidime and meropenem nonsusceptibility in *P. aeruginosa*, and cefoxitin resistance in *S. aureus*. Overall, sensitivities exceeded 98% for the majority of organism–antibiotic pairs, with specificities ranging from 88.4% to 100%. Categorical agreement was high at 97.9%, ranging from 88.8% to 100% across organism groups. The overall rates of major error and very major error were very low, at 0.2% and 0.04%, respectively, and ranged from 0% to 1.5% and 0% to 0.04%, respectively, across organism groups. In conclusion, dDDT is a reliable and expedient method for detecting antibiotic nonsusceptibility, making it a valuable tool for the timely management of bloodstream infections caused by resistant organisms.

## 1. Introduction

Bloodstream infections (BSIs) are associated with significant morbidity and mortality and places a heavy burden on healthcare systems [1,2]. Among the pathogens responsible, *Staphylococcus aureus* and *Escherichia coli* are the leading causes of BSIs [3]. Other notable Gram-negative organisms include *Klebsiella pneumoniae* complex, *Pseudomonas aeruginosa,* and *Enterobacter cloacae* complex. Common Gram-positive organisms apart from *S. aureus* are *Enterococcus faecalis*, *Enterococcus faecium,* and *Streptococcus agalactiae* [3].

A key emerging challenge in managing BSIs is the rise in multidrug-resistant organisms (MDROs), which severely limits effective treatment options [4,5]. In 2021, it was estimated that 1.1 million deaths globally were attributed to bacterial antimicrobial resistance (AMR), and this was forecasted to increase to 1.9 million in 2050 [6]. Methicillin-resistant *S. aureus* (MRSA) remains a major concern, accounting for 37% of *S. aureus* bacteremia cases globally between 1997 and 2016 [7]. Among Enterobacterales, resistance to third-generation cephalosporin, primarily driven by extended-spectrum β-lactamases (ESBLs, mainly CTX-M) and AmpC cephalosporinases, is frequently observed [8,9,10,11]. According to a global surveillance program, the proportion of ESBL-producing *E. coli* among all *E. coli* BSIs was 29% in 2018–2019, and was up to 53.1% in China [12]. In comparison, infections caused by carbapenemase-producing Enterobacterales (CPE), which include serine carbapenemases like KPC, OXA-48, as well as metallo-β-lactamases like NDM-1, are less prevalent but are increasing and represent a growing concern [13,14,15]. Globally, other emerging MRDOs, such as carbapenem-resistant *P. aeruginosa*, are also a cause for concern [16,17]. WHO Bacterial Priority Pathogens List encompasses these resistant phenotypes, underscoring their global burden and the urgent need for effective interventions [18].

Effective treatment of BSIs hinges on timely and appropriate antibiotic therapy. Patients who receive inappropriate empirical antibiotics face significantly higher mortality and recurrence rates of bacteremia compared to those receiving appropriate treatment [19,20]. Therefore, accurate and rapid antibiotic susceptibility testing is vital for optimizing treatment outcomes. Detecting resistance to antibiotics commonly prescribed as first-line treatment for sepsis is especially important. Direct disk diffusion testing (dDDT) stands as one of the most widely used rapid susceptibility testing methods, with the majority of European laboratories utilizing this approach [21]. Unlike the standard susceptibility testing method that uses a suspension from bacterial colonies as the inoculum, dDDT utilizes an aliquot from positive blood culture broths as the inoculum, providing results one day earlier.

This study aims to assess the effectiveness of dDDT in predicting clinically important and commonly encountered antimicrobial resistance phenotypes. Frequently isolated Gram-negative and Gram-positive bacteria from blood cultures are included. The aim was also to address the diagnostic gap of dDDT for certain Gram-positive organisms, such as *Enterococcus* species and β-hemolytic streptococci.

## 2. Methods

### 2.1. Data Collection

A retrospective study was conducted using data from Kwong Wah Hospital (KWH), a regional hospital in Hong Kong with more than 1400 beds providing acute services. Information including patient identifiers, culture dates, organisms, and susceptibility results from all blood cultures between January 2021 and December 2023 was extracted. The blood cultures were obtained from inpatients in various departments, including Medicine and Geriatrics, Accident and Emergency, Intensive Care Unit, Surgery, Orthopedics and Traumatology, Neurosurgery, Obstetrics and Gynecology, and Pediatrics and Adolescent Medicine. The standard disk diffusion testing results were sourced from the laboratory information system, while dDDT results were retrieved from hardcopy laboratory forms. Blood cultures were collected using BD BACTEC™ Plus Aerobic/F and BD BACTEC™ Lytic/10 Anaerobic/F culture vials, which were then incubated in the automated BD BACTEC™ FX blood culture system (Becton Dickinson, Franklin Lakes, NJ, USA). From positive cultures, the bacteria were identified using MALDI-ToF MS (Microflex LT; Bruker Diagnostics, Bremen, Germany). Bacterial isolates categorized as Enterobacterales, *P. aeruginosa*, *S. aureus*, *Enterococcus* spp., or β-hemolytic streptococci were included. These bacteria were selected because they are frequently isolated from blood cultures and represent the leading causes of bacteremia. They are also key pathogens associated with antimicrobial resistance, which would benefit from early susceptibility testing results to guide effective treatment. Isolates were eligible if documented results for both dDDT and reference disk diffusion testing (rDDT) were accessible for all antibiotics in the designated panels. Polymicrobial blood cultures and duplicate cultures with the same organism from the same patient within 14 days of the initial culture were excluded.

### 2.2. Direct and Colony Antimicrobial Susceptibility Testing

Direct disk diffusion testing was performed by mixing 0.5 mL of the positive blood culture broth into 2 mL of sterile 0.85% saline. Following mixing, a sterile cotton swab was then used to inoculate a 90 mm Mueller–Hinton agar (MHA) plate by immersing the tip into the suspension, then rubbing the swab over the entire surface of the plate using an automatic plate rotator (Electrotek, Tamworth, UK). Based on the Gram stain results from positive blood culture bottles, corresponding panels of antibiotics were chosen, and antimicrobial disks were applied onto the inoculated plates using a disk dispenser. The same swab was then used to inoculate a purity plate. The plates were incubated at 35 °C for 20 to 24 h under 5% CO_2_ for streptococci or for 16 to 18 h in ambient air for all other organisms. Zone sizes were then measured and interpreted according to the Clinical and Laboratory Standards Institute (CLSI) 2021 guidelines. On the next day, the disk diffusion method was performed using bacterial colonies according to the CLSI and used as the reference method. All dDDT and rDDT results were interpreted according to the CLSI 2021 guidelines and categorized as susceptible (S), intermediate (I), or resistant (R) (Table S1) [22]. *Salmonella* results were analyzed separately from other members of Enterobacterales, as the CLSI recommends a different panel of antibiotics for susceptibility testing. Organism–antibiotic pairs with known intrinsic resistance were classified as resistant, regardless of the testing results [22].

In Enterobacterales, the production of ESBL was identified using the CLSI combined disk diffusion method, by using ceftazidime and cefotaxime disks, alone or in combination with clavulanate [22]. An ESBL-positive isolate was classified as resistant to ceftriaxone, ceftazidime, and cefepime. Additionally, if an Enterobacterales isolate was resistant to ceftriaxone and ceftazidime but susceptible to cefepime, and ESBL testing was negative, the isolate was designated as AmpC β-lactamase positive [11]. Enterobacterales isolates that exhibited nonsusceptibility to meropenem underwent further testing for carbapenemase production using a lateral flow immunoassay (NG-Test CARBA-5, NG Biotech, Guipry, France), which can detect KPC, NDM, IMP, VIM, and OXA-48-like enzymes [23].

### 2.3. Data Analysis

The prevalence of nonsusceptibility was graded as follows: rare (<0.1%), very low (0.1–<1%), low (1–10%), moderate (11–20%), high (21–50%), very high (51–70%), and extremely high (≥71%) [24]. The diagnostic performance of dDDT was evaluated based on its ability to detect antibiotic nonsusceptibility in patients with bacteremia, using rDDT as a reference. Nonsusceptibility was defined by both intermediate and resistant results. True positives (TPs) were cases where both dDDT and rDDT indicated nonsusceptibility, while true negatives (TNs) were cases with both testing methods showing susceptibility. False positives (FPs) referred to cases where dDDT was nonsusceptible but rDDT was susceptible. Contrarily, false negatives (FNs) were isolates that dDDT classified as susceptible while rDDT indicated nonsusceptibility.

Sensitivity, specificity, positive predictive values (PPVs), and negative predictive values (NPVs) were calculated using standard definitions. Sensitivity was calculated as the proportion of TP out of all TP and FN, while specificity was the proportion of TN out of all TN and FP. PPV and NPV were calculated as TP ÷ (TP + FP) and TN ÷ (TN + FN), respectively. The 95% confidence intervals (CIs) were calculated an online statistical calculation tool (MedCalc, Ostend, Belgium) [25].

Categorical agreement (CA), minor error (mE), major error (ME), and very major error (VME) rates were calculated and expressed in percentages according to published guidance [26]. CA was defined by identical results between dDDT and rDDT. ME referred to false resistance (resistant by dDDT and susceptible by rDDT), whilst VME indicated false susceptibility (susceptible by dDDT and resistant by rDDT). All other mismatches between dDDT and rDDT that differed by one susceptibility category were classified as mE. The rates of CA and mE were calculated as the number of CA or mE divided by the total number of isolates tested. The rates of ME and VME were calculated by dividing the number of ME or VME results by the total number of isolates classified as susceptible or resistant by rDDT, respectively.

## 3. Results

A total of 3271 positive blood cultures were retrospectively identified from January 2021 to December 2023. Of these, 1798 cultures were excluded from analysis including 504 cultures with polymicrobial growths, 794 cultures with incomplete dDDT or rDDT data, and 500 cultures with duplicated blood cultures with the same organism within 14 days of the initial culture. This left 1473 positive blood cultures for analysis, which included Enterobacterales (excluding *Salmonella*) (*n* = 1000), *Salmonella* spp. (*n* = 41), *P. aeruginosa* (*n* = 34), *S. aureus* (*n* = 272), β-hemolytic streptococci (*n* = 89), and *Enterococcus* spp. (*n* = 37). A total of 9754 organism–antibiotic test pairs were analyzed.

The diagnostic performance of dDDT and the prevalence of antibiotic nonsusceptibility are summarized in Table 1. Significant variations were observed in nonsusceptibility rates across different antibiotics. In Enterobacterales, the nonsusceptibility rate was extremely high for ampicillin (83.6%) and high (22.5−31.2%) for cefuroxime, ceftriaxone, ceftazidime, cefepime, amoxicillin-clavulanate, and levofloxacin, but very low (0.1%) for meropenem. In *Salmonella*, the nonsusceptibility rate for ceftriaxone was high (22.0%), while all isolates were susceptible to meropenem. Among the 269 Enterobacterales isolates resistant to ceftriaxone, 257 were attributed to ESBL production, 5 to due to AmpC β-lactamase production, and 1 to carbapenemase production. In *S. aureus*, the nonsusceptibility rates were high for cefoxitin (43.4%) and levofloxacin (36.3%) and moderate for erythromycin (17.7%) and gentamicin (18.4%). Among β-hemolytic streptococci, the nonsusceptibility rate was high for erythromycin (43.8%) and low for levofloxacin (3.4%), while all isolates were susceptible to penicillin, ceftriaxone, and vancomycin (Table 1).

In Enterobacterales, dDDT demonstrated a high PPV for detecting nonsusceptibility, ranging from 96.6% to 100% for ampicillin, cefuroxime, ceftriaxone, ceftazidime, cefepime, and meropenem (Table 1). On the other hand, amoxicillin-clavulanate and levofloxacin had relatively lower PPVs of 85% and 89%, respectively. In *Salmonella*, all nine isolates with ceftriaxone nonsusceptibility from ESBL production were correctly detected by dDDT. All cefoxitin resistance in the 118 *S. aureus* isolates was correctly detected by dDDT. However, one cefoxitin-susceptible isolate was incorrectly flagged as cefoxitin-resistant by dDDT, resulting in a PPV of 99.2%. Erythromycin and gentamicin had lower PPVs at 67.6% and 87.7%, respectively. For β-hemolytic streptococci, the PPV of dDDT for erythromycin was 90.7%, while levofloxacin showed the lowest predictive value among all organism–antibiotic pairs at 23.1%. For *Enterococcus* spp., ampicillin demonstrated a PPV of 100%. The NPVs of dDDT for all the organism–drug pairs were high, ranging from 99.5% to 100% (Table 1). Overall, dDDT exhibited a sensitivity exceeding 98% for all organism-antibiotic pairs, except in the case of *P. aeruginosa* where one meropenem-intermediate result was reported as meropenem-susceptible. The specificities were high overall, except for erythromycin (89.7%) in *S. aureus* and for both erythromycin (92.0%) and levofloxacin (88.4%) in β-hemolytic streptococci. Comparable performance parameters were observed across the main groups of Enterobacterales, including *E. coli*, *Klebsiella*, *Proteus*, *Citrobacter*, *Enterobacter*, and *Serratia* (Table S2).

**Figure 1:**
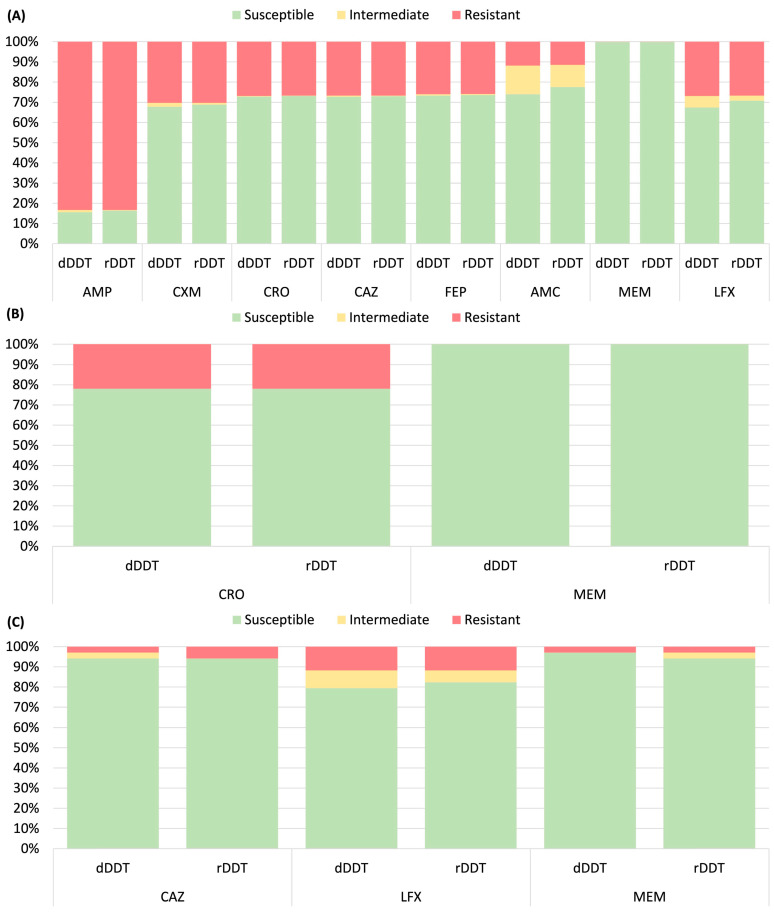
Comparison of antimicrobial susceptibility obtained by direct disk diffusion testing (dDDT) and reference disk diffusion testing (rDDT). Results for blood culture isolates of (**A**) Enterobacterales other than *Salmonella* (*n* = 1000), (**B**) *Salmonella* (*n* = 41), and (**C**) *P. aeruginosa* (*n* = 34). Abbreviations: AMC, amoxicillin-clavulanate; AMP, ampicillin; CAZ, ceftazidime; CRO, ceftriaxone; CXM, cefuroxime; FEP, cefepime; LFX, levofloxacin; MEM, meropenem.

**Figure 2:**
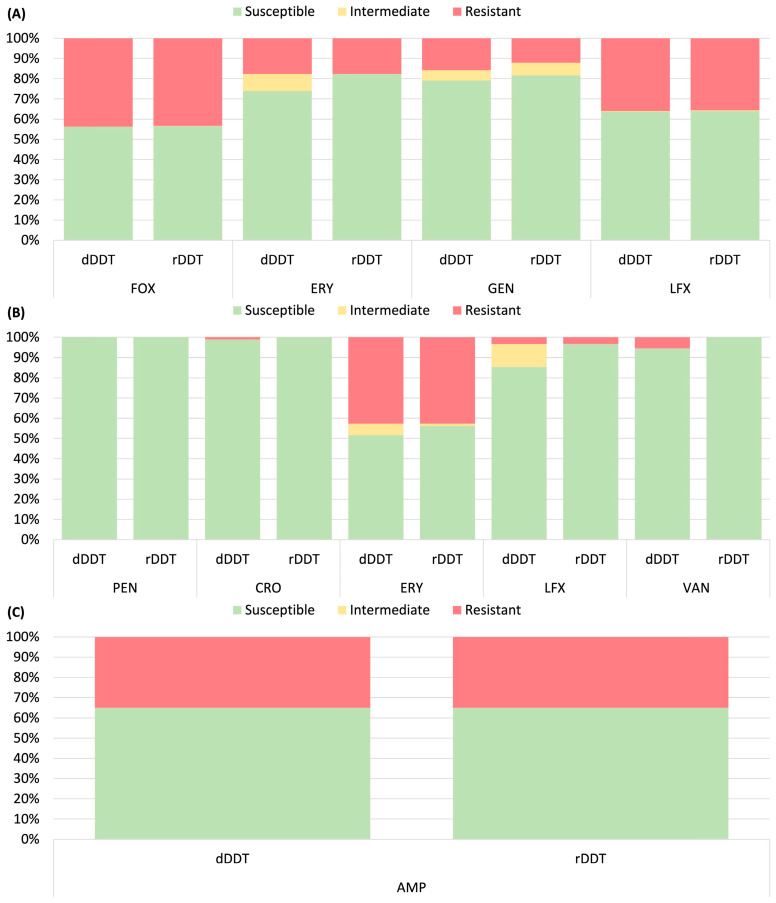
Comparison of antimicrobial susceptibility obtained by direct disk diffusion testing (dDDT) and reference disk diffusion testing (rDDT). Results for blood culture isolates of (**A**) *S. aureus* (*n* = 272), (**B**) β-hemolytic streptococci (*n* = 89), and (**C**) *Enterococcus* spp. (*n* = 37). Abbreviations: AMP, ampicillin; CRO, ceftriaxone; ERY, erythromycin; FOX, cefoxitin; GEN, gentamicin; LFX, levofloxacin; PEN, penicillin; VAN, vancomycin.

Figure 1 and Figure 2 compared dDDT and rDDT results by susceptibility categories across all tested organism–antibiotic pairs, illustrating a high categorical agreement between the two methods for the majority of the organism–drug pairs. Relatively lower categorical agreements were observed for amoxicillin-clavulanate (94.3%) and levofloxacin (95.9%) in Enterobacterales, erythromycin (91.5%) and gentamicin (93.8%) in *S. aureus*, and erythromycin (93.3%), levofloxacin (88.8%), and vancomycin (94.4%) in β-hemolytic streptococci (Table S3). Among the 8000 organism–antibiotic pairs in Enterobacterales, there were 128 mEs, 6 MEs, and 1 VME (Table 2 and Table S3). A higher mE rate was observed for amoxicillin-clavulanate (5.7%) and levofloxacin (4.0%). For *Salmonella*, all 82 organism–antibiotic pairs tested achieved 100% CA. For *P. aeruginosa*, 102 organism–antibiotic pairs were tested, with 3 mEs (2.9%) but no ME or VME.

In *S. aureus*, 40 out of 1088 organism–antibiotic pairs showed mEs, primarily due to erythromycin and gentamicin. There were 3 MEs (2 gentamicin and 1 cefoxitin) but no VMEs (Table 2). For β-hemolytic streptococci, 445 organism–antibiotic pairs were tested, with 16 mEs, predominantly due to levofloxacin. There were 6 MEs, in which vancomycin accounted for 5, with a ME rate of 5.6% and ceftriaxone accounted for 1, with a ME rate of 1.1%. No VMEs were observed for β-hemolytic streptococci. For *Enterococcus* spp., only ampicillin was tested, and all 37 isolates demonstrated 100% CA.

## 4. Discussion

Our data demonstrated a high PPV and specificity of dDDT in detecting nonsusceptibility in commonly isolated bacteria from blood cultures. The findings are applicable to critically important, resistance phenotypes that are prevalent among Enterobacterales (ceftriaxone, ceftazidime, cefepime, meropenem), *P. aeruginosa* (ceftazidime, meropenem), and *S. aureus* (cefoxitin). A group of Infectious Disease Physicians identified the rapid detection of drug-resistant aerobic Gram-negative bacilli and MRSA as the most pressing diagnostic needs [27]. Effective antibiotics is key in treatment, yet inappropriate empirical antibiotics are not uncommonly prescribed and was noted in a significant proportion of patients with bloodstream infections caused by CPE and ESBL-producing Enterobacterales [28]. Early and accurate antimicrobial susceptibility results are therefore crucial in sepsis treatment. dDDT results can be used to provide accurate and actionable results one day earlier. This enables the escalation of antibiotics to cover nonsusceptible isolates or narrowing down the spectrum of treatment to reduce the selection for antibiotic resistance. When integrated with antibiotic stewardship programs, studies have demonstrated rapid blood culture diagnostics as useful and cost-effective, with some showing mortality benefit [29,30].

The overall rates of major error and very major error were very low across all the organism and antibiotic groups. The organism and drug distribution of these errors did not suggest any predilection of a specific group of organisms or class of antibiotics. These errors may be due to factors such as excessive or insufficient inoculum or interference with drug diffusion from the presence of blood. An isolated case of false resistance to cefoxitin in *S. aureus* was encountered. In *S. aureus*, cefoxitin is utilized as a proxy for detecting *mec* gene-mediated β-lactam resistance, with breakpoints indicating susceptibility or resistance. In the absence of an intermediate category, isolates with zone sizes near the breakpoints may be misclassified, leading to false resistance or false susceptibility [22,31]. To improve the accuracy of detecting cefoxitin susceptibility in *S. aureus*, one practical solution is to subject isolates with zone sizes near the breakpoints to PBP2a assays [32,33]. Our findings of very low major error and very major error rates align with previous findings in the literature on dDDT for Gram-negative organisms, but contrast with observations for β-hemolytic streptococci and *Enterococcus* species, where recommendations were made against using dDDT due to unacceptable high error rates [34,35]. Although our data demonstrated promising results with low error rates for β-hemolytic streptococci, it should be noted that five cases of false resistance to vancomycin were observed. Given the exceedingly rare occurrence of vancomycin resistance in this group of organisms, the dDDT results should be confirmed before any definitive action is taken.

Direct disk diffusion method for positive blood cultures is simple to execute and does not necessitate any additional instruments. The diagnostic performance that we obtained was considered to be acceptable using the current standards [26]. Both CLSI and European Committee on Antimicrobial Susceptibility Testing (EUCAST) have developed rapid antimicrobial susceptibility testing methods based on the disk diffusion testing, which are read at shortened incubation times (4–10 h) [22,36,37,38,39]. The inhibition zone sizes change over incubation time, as the zone sizes of susceptible isolates grow larger whilst resistant isolates grow smaller. A report by CLSI revealed that results read at 6 h showed low categorical agreement when standard breakpoints were used [36]. In contrast, EUCAST breakpoints that were adapted to shortened incubation time yielded good performance in European laboratories [37]. To address this, both agencies developed time-specific breakpoints, and EUCAST introduced an area of uncertainty as a buffer to cope with technical variations. The CLSI developed breakpoints for Enterobacterales and *P. aeruginosa*, while EUCAST covered species-specific Enterobacterales (*E. coli*, *K. pneumoniae* complex and *Salmonella enterica*), *P. aeruginosa*, *Acinetobacter baumannii*, *S. aureus*, *E. faecalis*, *E. faecium,* and *Streptococcus pneumoniae*. A challenge observed with the rapid disk diffusion testing approach is that a considerable proportion of results could not be read after short incubation time due to hazy growth and poorly demarcated zone edges, particularly in relatively slow growing organisms like *P. aeruginosa* and Gram-positive bacteria [36,38]. This limitation may also be one important reason why breakpoints for β-hemolytic streptococci have not been developed in either guideline, as these organisms are known to be slow-growing and form small colonies. Although the rapid disk diffusion testing can greatly reduce the time to results, its implementation is challenging in laboratories that do not operate around the clock or lack automated systems for reading inhibition zone sizes at various time points. This is why we did not evaluate dDDT at a shortened incubation time.

In our study, dDDT was performed by diluting blood culture broths in a 1:4 ratio. BD Bactec blood culture bottles flagged positive by the BACTEC FX system contains an average of 6.9 × 10^8^ CFU/mL of bacteria; therefore, dilution aims to achieve a bacterial concentration similar to the 0.5 McFarland standard (1.5 × 10^8^ CFU/mL) used in rDDT [36]. In addition, blood components in the inoculum may interfere with disk diffusion testing of β-lactams; hence, dilution may reduce this interference [36]. Both CLSI and EUCAST recommend using undiluted blood culture broth for rapid antimicrobial susceptibility testing, but some studies have employed different dilution protocols and achieved low major error and very major error rates. These protocols include adding 1–3 drops of blood culture broth (depending on Gram stain results) into 1.8 mL of saline, adding 40 or 200 μL of broth (depending on Gram stain results) to 1 mL of saline, or diluting 0.1 mL of broth into 5 mL of saline [34,35,40]. Results are typically read at 16–20 h, which contrasts with the shorter incubation times of the rapid testing methods recommended by the CLSI and EUCAST (4–10 h), where significantly higher inoculum concentrations are used. Dilution of blood culture broth involves an additional step which is more time-consuming than using the broth directly. However, our dilution protocol produced dDDT results that are interpretable and reliable across all tested organism–antibiotic pairs, including Gram-positive organisms. Another approach involves two-step centrifugation and resuspension of broth to achieve a 0.5 McFarland standard, but this method is comparatively more time-consuming and labor-intensive [41].

dDDT can be seamlessly integrated into the workflow of processing positive blood culture bottles, as it is performed alongside with routine Gram staining and agar plate inoculation. It is also affordable and easily accessible, making it the most widely adopted direct antimicrobial susceptibility method in European laboratories, and a practical option in resource-limited settings [21]. Nonetheless, dDDT faces several diagnostic challenges, one of which is mixed infections. Although most blood cultures are monomicrobial, polymicrobial infections can interfere with dDDT results. To minimize the risk of ambiguous or inaccurate results, careful Gram staining should be performed to detect mixed infections. If present, dDDT results should be interpreted with caution or disregarded. We did not analyze the ability of dDDT to detect heteroresistance, which is known to be difficult to identify by disk diffusion susceptibility testing [42]. For indeterminate dDDT results, additional confirmatory tests should be performed, such as PBP2a assays for cefoxitin susceptibility in *S. aureus* [32,33]. These factors should be carefully considered when implementing dDDT in clinical practice.

The strengths of our study include a relatively large sample size compared to that reported in the previous literature, which encompassed the most common pathogens isolated from blood cultures. However, several limitations should be noted. First, this was a retrospective single-center study. Second, most dDDT results were reported in categories with no zone diameters, which restricted our ability to confirm the accuracy of susceptibility interpretations. Lastly, we did not include piperacillin–tazobactam, one of the most commonly used empirical antibiotics in our analysis. This was due to revision of the CLSI breakpoints in 2022 for Enterobacterales and 2023 for *P. aeruginosa*, which were within our study period.

In conclusion, our study demonstrated a high predictive value of dDDT on identifying nonsusceptibility to commonly used antibiotics in BSIs, with overall very low major error and very major error rates. The fast turnaround time of dDDT can aid clinicians in patient management and antibiotics stewardship to improve patient outcomes. Though dDDT remains manual and labor-intensive compared to innovative and commercial techniques, it is an easily accessible and affordable diagnostic tool and has the potential to be widely implemented in routine clinical microbiology laboratories.

## Figures and Tables

**Table 1:** Diagnostic performance parameters of direct disk diffusion testing (dDDT) for detection of antibiotic nonsusceptibility in patients with bacteremia.

Organism and Antibiotic	% (95% Confidence Interval)				
	PPV	NPV	Sensitivity	Specificity	Prevalence
Enterobacterales ^a^ (*n* = 1000)					
Ampicillin	99.1 (98.2, 99.5)	100 (97.7, 100)	100 (99.6, 100)	95.1 (90.6, 97.9)	83.6 (81.2, 85.8)
Cefuroxime	96.6 (94.0, 98.1)	99.9 (99.0, 100)	99.7 (98.2, 100)	98.4 (97.2, 99.2)	31.2 (28.3, 31.2)
Ceftriaxone	98.9 (96.7, 99.6)	100 (99.5, 100)	100 (98.6, 100)	99.6 (98.8, 99.9)	26.9 (24.2, 29.8)
Ceftazidime	98.9 (96.7, 99.6)	100 (99.5, 100)	100 (98.6, 100)	99.6 (98.8, 99.9)	27.0 (24.3, 29.9)
Cefepime	98.5 (96.1, 99.4)	99.9 (99.0, 100)	99.6 (97.9, 100)	99.5 (98.6, 99.9)	26.4 (23.7, 29.3)
Meropenem	100 (2.5, 100)	100 (99.6, 100)	100 (2.5, 100)	100 (99.6, 100)	0.1 (0.0, 0.6)
AMC	85.0 (80.7, 88.5)	99.5 (98.6, 99.8)	98.2 (95.5, 99.5)	95.0 (93.2, 96.4)	22.5 (20.0, 25.2)
Levofloxacin	89.0 (85.4, 91.7)	99.7 (98.8, 99.9)	99.3 (97.6, 99.9)	94.9 (93.0, 96.4)	29.2 (26.4, 32.1)
*Salmonella* (*n* = 41)					
Ceftriaxone	100 (66.4, 100)	100 (89.1, 100)	100 (66.4, 100)	100 (89.1, 100)	22.0 (10.6, 37.6)
Meropenem	-	100 (91.4, 100)	-	100 (91.4, 100)	0.0 (0.0, 8.6)
*P. aeruginosa* (*n* = 34)					
Ceftazidime	100 (15.8, 100)	100 (89.1, 100)	100 (15.8, 100)	100 (89.1, 100)	5.9 (0.7, 19.7)
Meropenem	100 (2.5, 100)	97.0 (88.9, 99.2)	50.0 (1.3, 98.7)	100 (89.1, 100)	5.9 (0.7, 19.7)
Levofloxacin	85.7 (46.7, 97.6)	100 (87.2, 100)	100 (54.1, 100)	96.4 (81.7, 99.9)	17.7 (6.8, 34.5)
*S. aureus* (*n* = 272)					
Cefoxitin	99.2 (94.4, 99.9)	100 (97.6, 100)	100 (96.9, 100)	99.4 (96.4, 100)	43.4 (37.4, 49.5)
Erythromycin	67.6 (58.6, 75.5)	100 (98.2, 100)	100 (92.6, 100)	89.7 (85.0, 93.4)	17.7 (13.3, 22.7)
Gentamicin	87.7 (77.5, 93.7)	100 (98.3, 100)	100 (92.9, 100)	96.9 (93.6, 98.7)	18.4 (14.0, 23.5)
Levofloxacin	99.0 (93.3, 99.9)	100 (97.9, 100)	100 (96.3, 100)	99.4 (96.8, 100)	36.3 (30.6, 42.3)
BHS (*n* = 89)					
Penicillin	-	100 (95.9, 100)	-	100 (95.9, 100)	0.0 (0.0, 4.1)
Ceftriaxone	-	100 (95.9, 100)	-	98.9 (93.9, 100)	0.0 (0.0, 4.1)
Erythromycin	90.7 (79.2, 96.2)	100 (92.3, 100)	100 (91.0, 100)	92.0 (80.8, 97.8)	43.8 (33.3, 54.8)
Levofloxacin	23.1 (14.4, 35.0)	100 (95.3, 100)	100 (29.2, 100)	88.4 (79.7, 94.3)	3.4 (0.7, 9.5)
Vancomycin	0.0	100 (95.7, 100)	-	94.4 (87.4, 98.2)	0.0 (0.0, 4.1)
*Enterococcus* spp. (*n* = 37)					
Ampicillin	100 (75.3, 100)	100 (85.8, 100)	100 (75.3, 100)	100 (85.8, 100)	35.1 (20.2, 52.5)

Abbreviations: AMC, amoxicillin-clavulanate; BHS, β-hemolytic streptococci; NPV, negative predictive value; PPV, positive predictive value. ^a^ Enterobacterales other than *Salmonella*; sensitivity, the proportion of nonsusceptible isolates that were identified as nonsusceptible by dDDT; specificity, the proportion of susceptible isolates that were identified as susceptible by dDDT; PPV, probability that nonsusceptiblity is present when nonsusceptibility is identified by dDDT; NPV, probability that nonsusceptiblity is absent when nonsusceptibility is not identified by dDDT.

**Table 2:** Summary of organism–antibiotic pairs with major error and very major error.

		No.			No.			%		
	Total	S	I	R	mE	MEs	VMEs	mE	ME	VME
Enterobacterales	8000	5531	157	2312	128	6 ^a^	1 ^d^	1.6	0.1	0.04
*Salmonella*	82	73	0	9	0	0	0	0	0	0
*P. aeruginosa*	102	92	3	7	3	0	0	2.9	0	0
*S. aureus*	1088	774	18	296	40	3 ^b^	0	3.7	0.4	0
BHS	445	403	1	41	16	6 ^c^	0	3.6	1.5	0
*Enterococcus* spp.	37	24	0	13	0	0	0	0	0	0
Total	9754	6897	179	2678	187	15	1	1.9	0.2	0.04

Abbreviations: BHS, β-hemolytic streptococci; S, susceptible; I, intermediate; ME, major error; R, resistant; VME, very major error; ^a^ including 1 ampicillin, 1 cefuroxime, 1 ceftriaxone, 2 ceftazidime, and 1 cefepime results.; ^b^ including 1 cefoxitin and 2 gentamicin results; ^c^ including 1 ceftriaxone and 5 vancomycin results; ^d^ including 1 levofloxacin result.

## Data Availability

The original contributions presented in this study are included in the article/Supplementary Material. Further inquiries can be directed to the corresponding author.

## Supplementary Materials

The following supporting information can be downloaded at: https://www.mdpi.com/article/10.3390/microorganisms13020398/s1, Table S1: Summary of breakpoints used in this study; Table S2: Diagnostic performance parameters of direct disk diffusion testing for detection of antibiotic nonsusceptibility in patients with bacteremia for the major groups of Enterobacterales; Table S3: Agreement of direct disk diffusion susceptibility test with reference disk diffusion method; Table S4: Summary of major errors and very major errors in this study.
