# Supplementary material for: Predictive Value of Direct Disk Diffusion Testing from Positive Blood Cultures for Detection of Antimicrobial Nonsusceptibility
Source: Microorganisms. 2025 Feb 12;13(2):398. doi: 10.3390/microorganisms13020398 (PMC11857890; doi:10.3390/microorganisms13020398)
Supplement: Supplementary file 1 [file microorganisms-13-00398-s001.zip › microorganisms-3451571-supplementary.pdf]

Supplementary file

Table S1. Summary of breakpoints used in this study

	Inhibition zone diameters (mm)[b]		
	Susceptible	Intermediate	Resistant
Enterobacterales[a]			
Ampicillin	≥ 17	14-16	≤ 13
Cefuroxime	≥ 18	15-17	≤ 14
Ceftriaxone	≥ 23	20-22	≤ 19
Ceftazidime	≥ 21	18-20	≤ 17
Cefepime	≥ 25	19-24	≤ 18
Meropenem	≥ 23	20-22	≤ 19
AMC	≥ 18	14-17	≤ 13
Levofloxacin	≥ 21	17-20	≤ 16
Salmonella			
Ceftriaxone	≥ 23	20-22	≤ 19
Meropenem	≥ 23	20-22	≤ 19
P. aeruginosa			
Ceftazidime	≥ 18	15-17	≤ 14
Meropenem	≥ 19	16-18	≤ 15
Levofloxacin	≥ 22	15-21	≤ 14
S. aureus			
Cefoxitin	≥ 22	-	≤ 21
Erythromycin	≥ 23	14-22	≤ 13
Gentamicin	≥ 15	13-14	≤ 12
Levofloxacin	≥ 19	16-18	≤ 15
BHS			
Penicillin	≥ 24	-	-
Ceftriaxone	≥ 24	-	-
Erythromycin	≥ 21	16-20	≤ 15
Levofloxacin	≥ 17	14-16	≤ 13
Vancomycin	≥ 17	-	-
Enterococcus spp.			
Ampicillin	≥ 17	-	≤ 16

Abbreviations: AMC, amoxicillin-clavulanate; BHS, beta-hemolytic streptococci

[a]Enterobacterales other than *Salmonella*

[b]CLSI M100 Edition 31

Table S2. Diagnostic performance parameters of direct disk diffusion testing (dDDT) for detection of antibiotic nonsusceptibility in patients with bacteremia for the major groups of Enterobacterales

	PPV	NPV	Sensitivity	Specificity	Prevalence
E. coli (n=679)					
Ampicillin	98.5 (97.2, 99.2)	100 (97.3, 100)	100 (99.3, 100)	94.4 (89.4, 97.6)	78.8 (75.5, 81.8)
Cefuroxime	96.0 (92.8, 97.8)	99.8 (98.4, 100)	99.6 (97.7, 100)	97.7 (95.9, 98.9)	35.1 (31.5, 38.8)
Ceftriaxone	98.7 (96.1, 99.6)	100 (99.2, 100)	100 (98.4, 100)	99.3 (98.1, 99.9)	33.3 (29.8, 37.0)
Ceftazidime	99.6 (97.0, 99.9)	100 (99.2, 100)	100 (98.4, 100)	99.8 (98.8, 100)	33.6 (30.0, 37.3)
Cefepime	99.1 (96.6, 99.8)	100 (99.2, 100)	100 (98.4, 100)	99.6 (98.4, 100)	33.3 (29.8, 37.0)
Meropenem	-	100 (99.5, 100)	-	100 (99.5, 100)	0.0 (0.0 to 0.5)
AMC	82.9 (76.9, 87.6)	99.2 (98.0, 99.7)	96.9 (92.3, 99.2)	95.3 (93.1, 96.9)	19.2 (16.3, 22.3)
Levofloxacin	91.2 (87.3, 94.0)	100 (99.1, 100)	100 (98.4, 100)	95.1 (92.7, 96.9)	33.6 (30.0, 37.3)
Klebsiella (n=201)					
Ampicillin	100 (98.2, 100)	-	100 (98.2, 100)	-	100 (98.2, 100)
Cefuroxime	96.6 (79.9, 99.5)	100 (97.9, 100)	100 (87.7, 100)	99.4 (96.8, 100)	13.9 (9.5, 19.5)
Ceftriaxone	100 (85.6, 100)	100 (97.9, 100)	100 (85.8, 100)	100 (97.9, 100)	11.9 (7.8, 17.2)
Ceftazidime	91.7 (73.5, 97.8)	100 (97.9, 100)	100 (84.6, 100)	98.9 (96.0, 99.9)	11.0 (7.0, 16.1)
Cefepime	95.2 (73.9, 99.3)	100 (98.0, 100)	100 (83.2, 100)	99.5 (97.0, 100)	10.0 (6.2, 15.0)
Meropenem	100 (2.5, 100)	100 (98.2, 100)	100 (2.5, 100)	100 (98.2, 100)	0.5 (0.01, 2.7)
AMC	87.5 (74.7, 94.3)	100 (97.7, 100)	100 (90.0, 100)	97.0 (93.1, 99.0)	17.4 (12.4, 23.4)
Levofloxacin	63.3 (49.1, 75.6)	99.4 (96.2, 99.9)	95.0 (75.1, 99.9)	93.9 (89.4, 96.9)	10.0 (6.2, 15.0)
Proteus (n=71)					
Ampicillin	100 (93.0, 100)	100 (83.2, 100)	100 (93.0, 100)	100 (83.2, 100)	71.8 (59.9, 81.9)
Cefuroxime	100 (80.5, 100)	100 (93.4, 100)	100 (80.5, 100)	100 (93.4, 100)	23.9 (14.6, 35.5)
Ceftriaxone	100 (79.4, 100)	100 (93.5, 100)	100 (79.4, 100)	100 (93.5, 100)	22.5 (13.5, 34.0)
Ceftazidime	100 (79.4, 100)	100 (93.5, 100)	100 (79.4, 100)	100 (93.5, 100)	22.5 (13.5, 34.0)
Cefepime	100 (79.4, 100)	100 (93.5, 100)	100 (79.4, 100)	100 (93.5, 100)	22.5 (13.5, 34.0)
Meropenem	-	100 (94.9, 100)	-	100 (94.9, 100)	0.0 (0.0, 5.1)
AMC	72.4 (58.2, 83.2)	100 (91.6, 100)	100 (83.9, 100)	84.0 (70.9, 92.8)	29.6 (19.3, 41.6)
Levofloxacin	97.7 (86.0, 99.7)	96.4 (79.5, 99.5)	97.7 (87.7, 99.9)	96.4 (81.7, 99.9)	60.6 (48.3, 72.0)
CES (n=35)					
Ampicillin	100 (90.0, 100)	-	100 (90.0, 100)	-	100 (90.0, 100)
Cefuroxime	100 (80.5, 100)	100 (81.5, 100)	100 (80.5, 100)	100 (81.5, 100)	48.6 (31.4, 66.0)
Ceftriaxone	100 (29.2, 100)	100 (89.1, 100)	100 (29.2, 100)	100 (89.1, 100)	8.6 (1.8, 23.1)
Ceftazidime	100 (29.2, 100)	100 (89.1, 100)	100 (29.2, 100)	100 (89.1, 100)	8.6 (1.8, 23.1)
Cefepime	50.0 (8.5, 91.5)	97.0 (88.9, 99.2)	50.0 (1.3, 98.7)	97.0 (84.2, 99.9)	5.7 (0.7, 19.2)
Meropenem	-	100 (90.0, 100)	-	100 (90.0, 100)	0.0 (0.0, 10.0)
AMC	100 (86.3, 100)	100 (69.2, 100)	100 (86.3, 100)	100 (69.2, 100)	71.4 (53.7, 85.4)
Levofloxacin	-	100 (89.7, 100)	-	97.1 (85.1, 99.9)	0.0 (0.0, 10.0)

Abbreviations: AMC, amoxicillin-clavulanate; CES, *Citrobacter*, *Enterobacter*, *Serratia*; NPV, negative predictive value; PPV, positive predictive value.

sensitivity = proportion of nonsusceptible isolates that were identified as nonsusceptible by dDDT; specificity = proportion of susceptible isolates that were identified as susceptible by dDDT; PPV = probability that nonsusceptibility is present when nonsusceptibility is identified by dDDT; NPV = probability that nonsusceptibility is absent when nonsusceptibility is not identified by dDDT.

Table S3. Agreement of direct disk diffusion susceptibility test with reference disk method

	No. of results from dDDT			Number of results (%)			
	S	I	R	CA	mE	ME	VME
Enterobacterales[a] (n=1000)							
Ampicillin	156	11	833	991 (99.1)	8 (0.8)	1 (0.6)	0 (0)
Cefuroxime	678	19	303	988 (98.8)	11 (1.1)	1 (0.1)	0 (0)
Ceftriaxone	728	3	269	994 (99.4)	5 (0.5)	1 (0.1)	0 (0)
Ceftazidime	727	6	267	995 (99.5)	3 (0.3)	2 (0.3)	0 (0)
Cefepime	733	7	260	995 (99.5)	4 (0.4)	1 (0.1)	0 (0)
Meropenem	999	0	1	1000 (100)	0 (0)	0 (0)	0 (0)
AMC	740	141	119	943 (94.3)	57 (5.7)	0 (0)	0 (0)
Levofloxacin	674	57	269	959 (95.9)	40 (4.0)	0 (0)	1 (0.4)
Subtotal	5435	244	2321	7865 (98.3)	128 (1.6)	6 (0.1)	1 (0.04)
Salmonella (n=41)							
Ceftriaxone	32	0	9	41 (100)	0 (0)	0 (0)	0 (0)
Meropenem	41	0	0	41 (100)	0 (0)	0 (0)	0 (0)
Subtotal	73	0	9	82 (100)	0 (0)	0 (0)	0 (0)
P. aeruginosa (n=34)							
Ceftazidime	32	1	1	33 (97.1)	1 (2.9)	0 (0)	0 (0)
Meropenem	33	0	1	33 (97.1)	1 (2.9)	0 (0)	0 (0)
Levofloxacin	27	3	4	33 (97.1)	1 (2.9)	0 (0)	0 (0)
Subtotal	92	4	6	99 (97.1)	3 (2.9)	0	0
S. aureus (n=272)							
Cefoxitin	153	0	119	271 (99.6)	0 (0)	1 (0.6)	0 (0)
Erythromycin	201	23	48	249 (91.5)	23 (8.5)	0 (0)	0 (0)
Gentamicin	215	14	43	255 (93.8)	15 (5.5)	2 (0.9)	0 (0)
Levofloxacin	173	1	98	270 (99.3)	2 (0.7)	0 (0)	0 (0)
Subtotal	742	38	308	1045 (96.0)	40 (3.7)	3 (0.4)	0 (0)
β-hemolytic streptococci (n=89)							
Penicillin	89	0	0	89 (100)	0 (0)	0 (0)	0 (0)
Ceftriaxone	88	0	1	88 (98.9)	0 (0)	1 (1.1)	0 (0)
Erythromycin	46	5	38	83 (93.3)	6 (6.7)	0 (0)	0 (0)
Levofloxacin	76	10	3	79 (88.8)	10 (11.2)	0 (0)	0 (0)
Vancomycin	84	0	5	84 (94.4)	0 (0)	5 (5.6)	0 (0)
Subtotal	383	15	47	423 (95.1)	16 (3.6)	6 (1.5)	0 (0)
Enterococcus spp. (n=37)							
Ampicillin	24	0	13	37 (100)	0 (0)	0 (0)	0 (0)
Total (n=1473)	6749	301	2704	9551 (97.9)	187 (1.9)	15 (0.2)	1 (0.04)

Abbreviations: AMC, amoxicillin-clavulanate; CA, categorical agreement; dDDT, direct disk diffusion testing; S, susceptible; I, intermediate; mE, minor error; ME, major error; R, resistant; VME, very major error

[a]Enterobacterales other than *Salmonella*

Table S4. Summary of major errors and very major errors in this study

Isolate	Species	Drug	Result by dDDT	Result by reference method	Type of error
913	*Klebsiella aerogenes*	Ceftazidime	R	S	ME
429	*Escherichia coli*	Ampicillin	R	S	ME
869	*Escherichia coli*	Cefuroxime	R	S	ME
869	*Escherichia coli*	Ceftriaxone	R	S	ME
869	*Escherichia coli*	Ceftazidime	R	S	ME
869	*Escherichia coli*	Cefepime	R	S	ME
216	*Proteus mirabilis*	Levofloxacin	S	R	VME
063	*Staphylococcus aureus*	Cefoxitin	R	S	ME
574	*Staphylococcus aureus*	Gentamicin	R	S	ME
582	*Staphylococcus aureus*	Gentamicin	R	S	ME
064	*Streptococcus agalactiae*	Ceftriaxone	R	S	ME
064	*Streptococcus agalactiae*	Vancomycin	R	S	ME
161	*Streptococcus group G*	Vancomycin	R	S	ME
718	*Streptococcus group G*	Vancomycin	R	S	ME
568	*Streptococcus group G*	Vancomycin	R	S	ME
699	*Streptococcus group G*	Vancomycin	R	S	ME

Abbreviations: dDDT, direct disk diffusion testing; S, susceptible; ME, major error; R, resistant; VME, very major error
